# A Novel Halophilic Lipase, LipBL, Showing High Efficiency in the Production of Eicosapentaenoic Acid (EPA)

**DOI:** 10.1371/journal.pone.0023325

**Published:** 2011-08-10

**Authors:** Dolores Pérez, Sara Martín, Gloria Fernández-Lorente, Marco Filice, José Manuel Guisán, Antonio Ventosa, María Teresa García, Encarnación Mellado

**Affiliations:** 1 Department of Microbiology and Parasitology, Faculty of Pharmacy, University of Seville, Seville, Spain; 2 Institute of Industrial Fermentation, Consejo Superior de Investigaciones Cientificas (CSIC), Madrid, Spain; 3 Department of Biocatalysis, Institute of Catalysis and Petrochemistry, Consejo Superior de Investigaciones Cientificas (CSIC), Autonomous University Campus, Cantoblanco, Madrid, Spain; University of Minnesota, United States of America

## Abstract

**Background:**

Among extremophiles, halophiles are defined as microorganisms adapted to live and thrive in diverse extreme saline environments. These extremophilic microorganisms constitute the source of a number of hydrolases with great biotechnological applications. The interest to use extremozymes from halophiles in industrial applications is their resistance to organic solvents and extreme temperatures. *Marinobacter lipolyticus* SM19 is a moderately halophilic bacterium, isolated previously from a saline habitat in South Spain, showing lipolytic activity.

**Methods and Findings:**

A lipolytic enzyme from the halophilic bacterium *Marinobacter lipolyticus* SM19 was isolated. This enzyme, designated LipBL, was expressed in *Escherichia coli*. LipBL is a protein of 404 amino acids with a molecular mass of 45.3 kDa and high identity to class C β-lactamases. LipBL was purified and biochemically characterized. The temperature for its maximal activity was 80°C and the pH optimum determined at 25°C was 7.0, showing optimal activity without sodium chloride, while maintaining 20% activity in a wide range of NaCl concentrations. This enzyme exhibited high activity against short-medium length acyl chain substrates, although it also hydrolyzes olive oil and fish oil. The fish oil hydrolysis using LipBL results in an enrichment of free eicosapentaenoic acid (EPA), but not docosahexaenoic acid (DHA), relative to its levels present in fish oil. For improving the stability and to be used in industrial processes LipBL was immobilized in different supports. The immobilized derivatives CNBr-activated Sepharose were highly selective towards the release of EPA versus DHA. The enzyme is also active towards different chiral and prochiral esters. Exposure of LipBL to buffer-solvent mixtures showed that the enzyme had remarkable activity and stability in all organic solvents tested.

**Conclusions:**

In this study we isolated, purified, biochemically characterized and immobilized a lipolytic enzyme from a halophilic bacterium *M. lipolyticus*, which constitutes an enzyme with excellent properties to be used in the food industry, in the enrichment in omega-3 PUFAs.

## Introduction

Lipases (E.C. 3.1.1.1) and carboxylesterases (E.C. 3.1.1.3) constitute the two classes of α/β-fold hydrolases, which are widely distributed in nature from bacteria to higher eucaryotes. Lipases are, by definition, enzymes that present the ability to hydrolyze long-chain acylglycerols (≥C_10_), whereas esterases hydrolyze ester substrates with short-chain fatty esters (≤C_10_) [1]. Lipases and esterases have been referred to collectively as “lipolytic enzymes” [2]. Indeed, they share a common structural motif which contains a serine residue within the consensus sequence G-X-S-X-G involved in the hydrolytic activity [3], [4].

There is an increasing interest for lipolytic enzymes and lipase-producing microorganisms as reactions catalysed by these enzymes show high selectivity and occur under mild conditions, with no requirement for added cofactors. They can also catalyse stereoselective and regioselective reactions, with applications in a variety of biotechnological fields such as food, dairy, detergent, pharmaceutical, agrochemical and oleochemical industries [1]. Since industrial processes are commonly carried out under harsh conditions, it would be of great importance to obtain lipases which retain their optimal activity at extremes of temperature, pH, different concentrations of salts and in the presence of organic compounds normally used in the industrial reactions as solvents [5]. In this sense, the lipases isolated from extreme microorganisms constitute an excellent alternative in the industrial processes [6].

Due to the physiological importance of the n-3 polyunsaturated fatty acids (n-3 PUFAs), various techniques have been used for concentrating these compounds, especially eicosapentaenoic acid (EPA: 20∶5n3) and docosahexaenoic acid (DHA: 22∶6n3). One of the most promising techniques is the use of lipase-catalyzed enzymatic hydrolysis reactions, which are generally more productive, compared to other concentration methods such as esterification and interesterification [7]. The utilization of lipases in this process is based on the fatty acids-specific selectivity that lipases exhibit toward saturated fatty acids (SFAs) and monounsaturated fatty acids (MUFAs), leaving n-3 PUFAs intact on the glyceride moiety [8]. Commercially available free lipases derived from microorganisms such as species of the genera *Candida*, *Aspergillus*, *Mucor* and others have been studied to be used in this reaction [9], [10], [11]. However, the use of pure soluble enzymes in chemical and biochemical reactions is expensive. Thus, in order to recover the enzyme, it is necessary to immobilize the enzyme in supports. Therefore, improvements in enzyme immobilization are a current focus of research in the fat and oil industries [12], [13]. A characteristic feature of lipases is their activation in the presence of hydrophobic interfaces (micelles of substrates, immiscible organic solvents, etc.) suffering critical conformational changes between a closed-inactive and an open-active structure [12], [13]. Recently, the interfacial activation of lipases on hydrophobic supports has been proposed as a simple alternative to selectively immobilize lipases from crude extracts at low ionic strength, where other water-soluble proteins are not adsorbed [14]. The active open form of the lipases is adsorbed on these supports via the big hydrophobic pocket conformed by the internal face of the lid and the areas surrounding the active centre. Furthermore, the hydrophobicity of the supports can permit the gradually adsorption of lipases to them [15].

In this study, we have purified and characterized the lipase LipBL in order to study its biochemical properties and we have obtained different LipBL derivatives that show a high efficiency in the production of fish oils enriched in PUFAs, but low enantioselectivity.

## Materials and Methods

### Microorganisms, media, inocula preparation and plasmids

*Marinobacter lipolyticus* SM19 (DSM 15157) was isolated from a saline soil in Cádiz, Spain (36° 27′ 06,90″N, 6° 12′ 07,60″O) [16].

This strain was grown in a saline medium (SW-7.5) with a total salt mixture concentration of 7.5% (w/v) supplemented with 0.5% (w/v) yeast extract [17]. The medium was adjusted to pH 7.4, prior to sterilization. *Escherichia coli* DH5α (Invitrogen) was used as the host for routine subcloning. All bacteria were cultivated at 37°C in an orbital shaker (New Brunswick Scientific) at 200 rpm. The cloning vector pBC KS (Phagemids) was used for subcloning of the enzyme-restricted DNA fragments and expression. All restriction enzymes were used as recommended by manufacturers.

### Cell fractionation of *M. lipolyticus* to determine the location of the enzyme

*M. lipolyticus* SM19 cells from 24 h cultures were harvested by centrifugation at 10000 *g* (Sorvall Evolution RC) for 20 min at 4°C. The culture supernatant was used to determine the extracellular enzyme activity. The pellet was washed in 25 mM phosphate buffer (pH 7.0). The cells were disrupted by ultrasonic treatment (Labsonic, Braun Biotech International) for 4 min (50%), and the cell debris was removed by centrifugation at 10000 *g* for 10 min at 4°C. The resulting supernatant was kept as the intracellular fraction and was stored at −20°C until use.

### Detection of lipase activity by zymogram

Zymographic analysis for lipolytic activity was performed in SDS-polyacrylamide gels using methylumbelliferyl (MUF)-butyrate as substrate [18]. After protein separation, SDS was removed from the gels by soaking them for 30 min in 2.5% (w/v) Triton X-100 at room temperature. The gels were then briefly washed in 50 mM phosphate buffer, pH 7.0, and covered by a solution of 100 µM MUF-butyrate in the same buffer. After UV illumination, fluorescent activity bands become visible in 30 seconds.

### Construction and screening of a genomic library of *M. lipolyticus*

To construct the library of *M. lipolyticus* we used the plasmid pHC79 as cloning vector [19] and *E. coli* XL1-Blue as the host strain. The genomic DNA was isolated from *M. lipolyticus* cells by CTAB method and was partially digested with *Eco*RI at 37°C. The resulting DNA fragments were electrophoresed in 0.6% agarose gel, and fragments with optimal size were ligated to pHC79, previously digested with *Eco*RI and dephosphorylated with bacterial alkaline phosphatase. The resultant plasmids were introduced into *E. coli* XL1-Blue. The library contained an estimated 20,000 *M. lipolyticus* clones in the *E. coli* host strain.

The screening of the library was performed using tributyrin plates. For that, the *E. coli* clones were grown in LB agar plates supplemented with ampicilin (150 µg ml^−1^) and 0.5% tributyrin. The positive clone cells were detected after 48 hours due to halo formation around the colonies.

### DNA manipulation

All DNA manipulations were carried out as described by Sambrook and Russell [20]. Restriction enzymes were obtained from Amersham Biosciences, United Kingdom.

The nucleotide sequence and its deduced amino acid sequence were analyzed using BLAST at NCBI [21].

### Culture conditions and preparation of samples for purification of LipBL

*E. coli* (pFP) cells from 24 h cultures grown at 37°C were harvested by centrifugation at 10000 *g* (Sorvall Evolution RC) for 10 min at 4°C. The culture supernatant was removed and the pellet was washed in 25 mM phosphate buffer (pH 7.0). The cells were disrupted by ultrasonic treatment (Labsonic, Braun Biotech International) for 4 min (50% power), and the cell debris was removed by centrifugation at 10000 *g* for 10 min at 4°C. The resulting supernatant was kept as the intracellular fraction and was stored at −20°C until use.

### Purification of the lipase LipBL

The purification of the enzyme from *E. coli* was carried out following a purification cascade combining cationic exchangers, ion metal-chelate affinity chromatography (IMAC) and hydrophobic supports, as follows: the crude extract from *E. coli* containing the recombinant lipase was incubated with sulfopropyl agarose (able to adsorb up to 30 mg ml^−1^ of proteins). The supernatant was offered to dextran-sulfate agarose and the enzyme bound with a yield of 95%. After washing of the enzyme-support complex with 100 mM NaCl in order to remove other proteins, the enzyme was eluted from the support with 350 mM NaCl. This solution was offered to highly activated iminodiacetic acid (IDA)-Cu supports (containing 20 µEqs of chelates per gram of 4% agarose gel), and the enzyme bound with a yield of 95.4%. The support was washed with 50 mM imidazole and then the enzyme was eluted by incubation in 100 mM imidazole and 150 mM NaCl. This supernatant was dialyzed 5 times against 10 mM phosphate buffer pH 7.0, using specific membranes from Medicell International Ltd, (Dialysis Tubing MWCO: 12–14,000 daltons; Size 6; 27/32′′; 21.5 mm). The dialyzed enzyme bound octyl-agarose with a yield of 90% and the complex was washed using 0.1% (w/v) Triton X-100 (more than 80% of the enzyme was retained on the support). The adsorbed lipase was eluted with 0.3% (w/v) Triton X-100.

### Protein determination

Protein concentration was measured according to Bradford [22], with a Bio-Rad kit and bovine serum albumin as the standard protein.

### SDS-PAGE analysis

SDS-PAGE electrophoresis was performed according to the method of Laemmli [23] in a BioRad electrophoretic unit using gels of 12% polyacrylamide with a separation zone of 9 cm×6 cm and a concentration zone of 5% polyacrylamide. The gels were stained following the Coomassie method. Molecular mass markers [LMW kit (14,400–97,000 Da)] were from Amersham.

### Peptide mass fingerprinting by MALDI-TOF

To identify LipBL in SDS-PAGE after the purification process, the digestion of trypsin, analysis of MALDI-TOF and identification of Peptide Mass Fingerprinting (PMF) was performed by the MBC (Molecular Biology Center, Madrid) using MASCOT search database [24].

### Lipase assays

#### Hydrolysis of *p-*NitroPhenylButyrate (*p*-NPB) [12]

This assay was performed by measuring continuously the increase in the absorbance at 348 nm produced by the release of *p*-nitrophenol in the hydrolysis of 0.4 mM *p-*NPB in 25 mM sodium phosphate buffer at pH 7.0 and 25°C. To initialize the reaction, 0.1 ml of lipase solution or suspension was added to 2.5 ml of substrate solution. One international unit (IU) of enzymatic activity was defined as the amount of enzyme necessary to hydrolyze 1 µmol of *p*-NPB per minute under the previously described conditions.

To investigate the substrate specificity of LipBL the enzyme activity was determined using the standard assay in the presence of 5 mM of the different *p*-nitrophenyl esters of various chain lengths: *p*-NP acetate (C2); *p*-NP butyrate (C4); *p*-NP caprylate (C8); *p-*NP decanoate (C10), *p-*NP laurate (C12) and *p*-NP myristate (C14). The experiments were carried out three times.

#### Hydrolysis of olive oil

For detection of olive oil hydrolysis, a plate assay containing olive oil [1–2% (w/v)] and rhodamine B [0.001% (w/v)] was performed [25]. The olive oil was prepared by ultrasonic treatment (Labsonic, Braun Biotech International) for 3 times during 10 min (100%). The medium was adjusted to pH 7.4, prior sterilization. Substrate hydrolysis causes the formation of orange fluorescent halos around bacterial colonies visible upon UV irradiation [25].

### Effect of temperature, pH and NaCl concentration on the hydrolytic activity of LipBL

The influence of temperature on p-NPB hydrolyzing activity was tested over the range from 5 to 90°C at pH 7.0. The thermostability profile of LipBL was measured by incubating the soluble enzyme at various temperatures (25°C to 60°C), followed by measuring residual activities at intervals of 30 min using the standard lipase assay. The influence of pH on p-NPB hydrolyzing activity was tested from pH 2.0 to 9.0 at 25°C. The influence of NaCl concentration on p-NPB-hydrolyzing activity was tested over the range of concentrations from 0 to 3 molar at 25°C and pH 7.0. In all cases, each analysis was performed in triplicate.

### Effect of organic solvents on the stability of LipBL

For study the effect of organic solvents in the stability of the pure soluble enzyme, LipBL was incubated in the presence of various organic compounds: dimethyl sulfoxide (DMSO), N,N-dimethyl formamide, methanol, acetonitrile, ethanol, diethylether, acetone, 1-propanol, 2-propanol (30% (v/v) in 25 mM sodium phosphate buffer at pH 7.0) and toluene and hexane (5% (v/v) in 25 mM sodium phosphate buffer at pH 7.0). Experiments were carried out at 25°C on a rotary shaker during 30 min and the lipolytic activity was measured using p-NP butyrate as substrate. A pure soluble enzyme preparation without any organic solvent was used as control. This experiment was performed in triplicate.

### Enzyme immobilization

In order to prepare the octyl agarose and dextran sulfate derivatives, an enzyme solution containing 6 mg of enzyme, at pH 7.0 and 25°C was mixed with 0.1 ml of support. At different times, samples of the supernatant, the support-enzyme suspension and enzyme solution incubated in the presence of the inert support were taken, and the hydrolytic activity and protein concentration were analyzed. The time was optimized according to the nature of the support and the enzyme.

The immobilization of LipBL on CNBr-activated support was performed for 15 min at 4°C to reduce the possibilities of getting a multipoint covalent attachment between the enzyme and the support. During the immobilization and further blocking of the support the suspension was submitted to continuous gentle stirring. The enzyme support immobilization was ended by incubating the support with 1 M ethanolamine at pH 8.0 for 2 h. Finally, the immobilized preparation was vacuum filtered using a sintered glass funnel and washed with abundant water, to eliminate the detergent. This immobilized enzyme was called CNBr-LipBL.

### Hydrolysis of fish oil

Hydrolysis of fish oil was performed in an organic and aqueous two-phase system. The procedure was as follows: 5 ml of hexane, 5 ml of 0.1 M phosphate buffer pH 6.0, and 0.5 ml of fish oil were placed in a reactor and preincubated at the conditions tested for 30 min. The reaction then was initiated by adding 0.3 g of lipase derivative and shaking at 150 rpm. pH-stat Mettler Toledo DL50 was used to stabilize pH during the reactions. The concentration of polyunsaturated free fatty acids was determined at various times by HPLC assay.

### Analysis of Polyunsaturated Free Fatty Acids (PUFAs) by HPLC and determination of hydrolysis conversion

After hydrolysis, aliquots of 0.1 ml of organic phase were obtained and dissolved in 0.8 ml of acetonitrile at different times. The unsaturated fatty acids produced were analyzed by RP-HPLC (Spectra Physic SP 100 coupled with an UV detector Spectra Physic SP 8450) using a kromasil C8 (15 cm×0.4 cm) column. Products were eluted at a flow rate of 1.0 ml/min using acetonitrile - 10 mM ammonium Tris buffer (70∶30, v/v) at pH 8.0. UV detection was performed at 215 nm. The retention times for the unsaturated fatty acids were: 9.4 min for eicosapentaenoic acid (EPA) and 13.5 min for docosahexaenoic acid (DHA).

The percentage of hydrolysis was calculated by the amount of EPA and DHA (PUFAs) released from their original content in the oil, considering that the PUFAs content in the fish oil was 30%. In an experiment, percent of peaks area was assumed as percent content of corresponding compound. PUFAs productivity (%) was calculated from the following equation: PUFAs (g min^−1^)  =  PUFAs (%) × fish oil (g ml^−1^), in which fish oil content was the weight of the fish oil present in the substrate mixture.

### Enzymatic hydrolysis of esters

The activities of different immobilized preparations of LipBL were analyzed in the hydrolysis reaction of different esters (dimethyl 3-phenylglutarate, methyl mandelate, 2-O-butyroyl mandelic acid, 4-phenyl-2-hydroxyethylbutyrate). Substrate 1 (dimethyl 3-phenylglutarate) (1 mM) was dissolved in 10 mM sodium phosphate at 25°C at different pH values and 0.25 g of immobilized preparation was added to 5 ml of this solution (Figure S1). Substrate (±)−4 (methyl mandelate) was dissolved in 3 ml of 10 mM sodium phosphate buffer with 2 mM of compound under different conditions (pH, temperature) and 0.6 g of immobilized preparation was added (Figure S2). Substrate (±)−5 (2-O-butyroyl mandelic acid) was dissolved in 3 ml of 10 mM sodium phosphate buffer with 0.5 mM of compound under different conditions (pH, temperature) and 0.5 g of immobilized preparation were added (Figure S3). Substrate (±)−7 (4-phenyl-2-hydroxyethylbutyrate) was dissolved in a 3 ml of 10 mM sodium phosphate buffer with 2 mM of compound under different conditions (pH, temperature) and 0.1 g of immobilized preparation was added (Figure S4).

During the reaction, the pH value was maintained constant using a pH-stat Mettler Toledo DL50 and the enzymatic activity was defined as µmol of substrate hydrolyzed per minute per mg of immobilized protein. The degree of hydrolysis was analyzed by reverse-phase HPLC (Spectra Physic SP 100 coupled with an UV detector Spectra Physic SP 8450). For these assays a Kromasil C18 (25×0.4 cm) column was used, mobile phase acetonitrile-10 mM ammonium phosphate buffer at pH 2.95 (35∶65, v/v) for compounds 1 and 5 (dimethyl 3-phenylglutarate and 2-O-butyroyl mandelic acid), (30∶70, v/v) for compound 4 (methyl mandelate), (40∶60, v/v) for compound 7 (4-phenyl-2-hydroxyethylbutyrate). UV detection was performed at 254 nm (compounds 4 and 5) and 270 nm (compound 7) (Figures S1, S2, S3 and S4).

### Determination of enantiomeric excess and enantioselectivity

The enantiomeric excess (ee) of the released acids (at conversions between 10 and 15%) was analyzed by Chiral Reverse Phase HPLC. The column used was a Chiracel OD-R, mobile phase, with an isocratic mixture of acetonitrile−NaClO4/HClO4 0.5 M, (5∶95 v/v) for compounds (±)−4 and (±)−5 and (20∶80 v/v) for compound (±)−7 with a final pH of 2.3, at a flow of 0.5 ml/min, In the case of compound 1, the mobile phase was an isocratic mixture of acetonitrile−10 mM ammonium phosphate (25∶75, v/v) at pH 3.0. UV detection was performed at 225 nm for all compounds. The enantiomeric ratio (*E*) was calculated using the equation reported by Chen et al. [26] (Figures S1, S2, S3 and S4).

### Nucleotide sequence accession number

The LipBL nucleotide sequence has been submitted to the EMBL database [EMBL:FR719924].

## Results

### Determination of the lipolytic activity of *M. lipolyticus*

**Figure 1:**
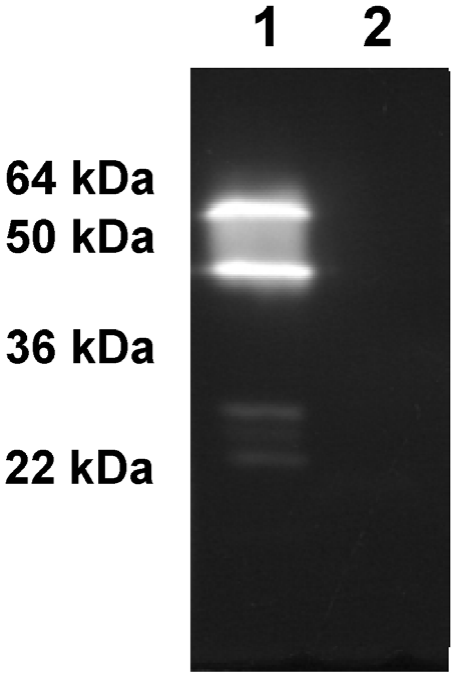
Zymographic analysis showing the lipolytic activity of *Marinobacter lipolyticus* on MUF-butyrate. Lane 1, intracellular fraction of *M. lipolyticus* SM19*;* Lane 2, extracellular fraction of *M. lipolyticus*.

*Marinobacter lipolyticus* SM19 is a moderately halophilic bacterium showing lipolytic activity in tributyrin plates [16]. To determine the number and the localization of lipolytic enzymes produced by *M. lipolyticus* SM19, the different cellular fractions of this bacterium were extracted and a zymographic analysis was performed as described in Materials and Methods. Lipolytic activity was identified in the intracellular fraction revealing the presence of at least one lipolytic enzyme, using MUF-butyrate as substrate (Figure 1).

### Isolation and characterization of *M. lipolyticus* lipolytic genes

In order to isolate lipolytic activity-encoding genes of *M. lipolyticus*, a genomic DNA library was constructed in *E. coli* using the cosmid pHC79 according to the method described in methods above. To screen the genomic DNA library the clones obtained were grown in LB medium with 0.5% of tributyrin and ampicillin (150 µg ml^−1^) and were incubated at 37°C during 48 hours. The most active clones against tributyrin (SM1–SM10) were selected for restriction analysis of the cosmids using the enzyme *Hin*dIII. The electrophoretic analysis showed similar restriction profiles for the isolated cosmids. The cosmid pSM9 was selected for further analysis. For that, the cosmid was digested with *Hin*dIII and subcloned in the vector pBC KS. The resultant clones were transformed in *E. coli* DH5α and cultured in LB medium supplemented with chloramphenicol (30 µg ml^−1^) and 0.5% of tributyrin. Only one clone showed a hydrolysis halo in the plates (pSR9.2 clone). The plasmid pSR9.2 contained a DNA insert of 6 kb, which was sequenced [27]. In this fragment, we identified 7 open reading frames (ORF) using the ORF Finder program of NCBI bioinformatic tools. Analysis of these ORFs showed that the ORF7 (1,215 bp), presented homology to other hydrolytic enzymes. The ORF7 was obtained from the plasmid pSR9.2 included in a 2.3 kb *Hin*dIII-*Sac*I fragment and cloned into the vector pBC KS. The plasmid obtained (5.4 kb) was named pFP and showed also lipolytic activity in tributyrin plates. Based on the high sequence similarity to hydrolytic enzymes, this gene was designated *lipBL* and the corresponding protein LipBL.

### Sequence analyses of LipBL

*lipBL* gene consisted of 1,215 bp, with an ATG start codon and TAA stop codon. The G+C content of the gene is 57.3% and encodes a predicted protein of 45.2 kDa, with a predicted pI value of 6.22.

Using BLAST data base for the sequence analysis, LipBL showed 91% similarity and 88% identity to *Marinobacter* sp. ELB17 β-lactamase, 83% similarity and 74% identity to *Marinobacter aquaeolei* VT8 β-lactamase, 68% similarity and 55% identity to *Rhodopseudomonas palustris* BisB5 β-lactamase and 58% similarity and 42% identity to the enzyme EstC isolated from environmental DNA (Table S1).

**Figure 2:**
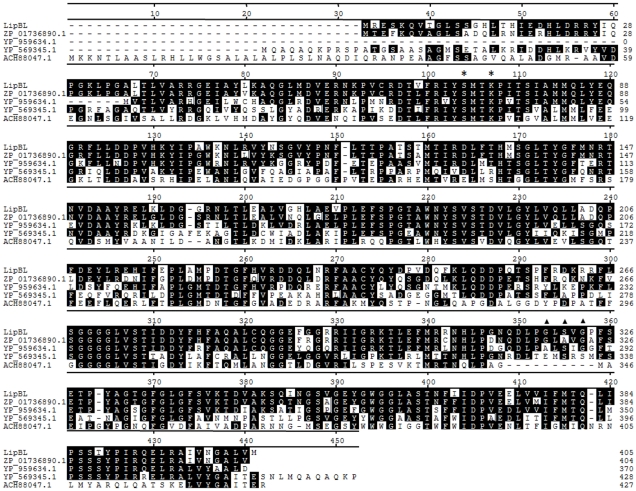
Alignment of LipBL to homologous proteins. *Marinobacter* sp. ELB17 β-lactamase (ZP_01736890.1), *Marinobacter aquaeolei* β-lactamase (YP_959634.1), *Rhodopseudomonas palustris* BisB5 beta-lactamase (YP_569345.1) and EstC isolated from environmental DNA (ACH88047.1). The conserved motif S-x-x-K (typical in class C β-lactamases and the family VIII of esterases) is indicated with asterisks and G-x-S-x-G motif (typical in lipases) is indicated with triangles.

Analysis of the amino acid sequence of lipBL revealed that the S_72_-M-T-K_75_ sequence is compatible with the S-x-x-K motif that is conserved in the class C β-lactamases, penicillin binding proteins (PBPs) [28], and carboxylesterases of family VIII [4]. Furthermore, the G-L-S-V-G sequence (amino acid positions 319–323), corresponding to a classical pentapeptide signature (G-x-S-x-G) motif was also observed at the C terminus of the LipBL primary structure (Figure 2). This motif contains the catalytic serine, responsible of the hydrolytic activity in other lipase families [29],

### Purification of LipBL

**Figure 3:**
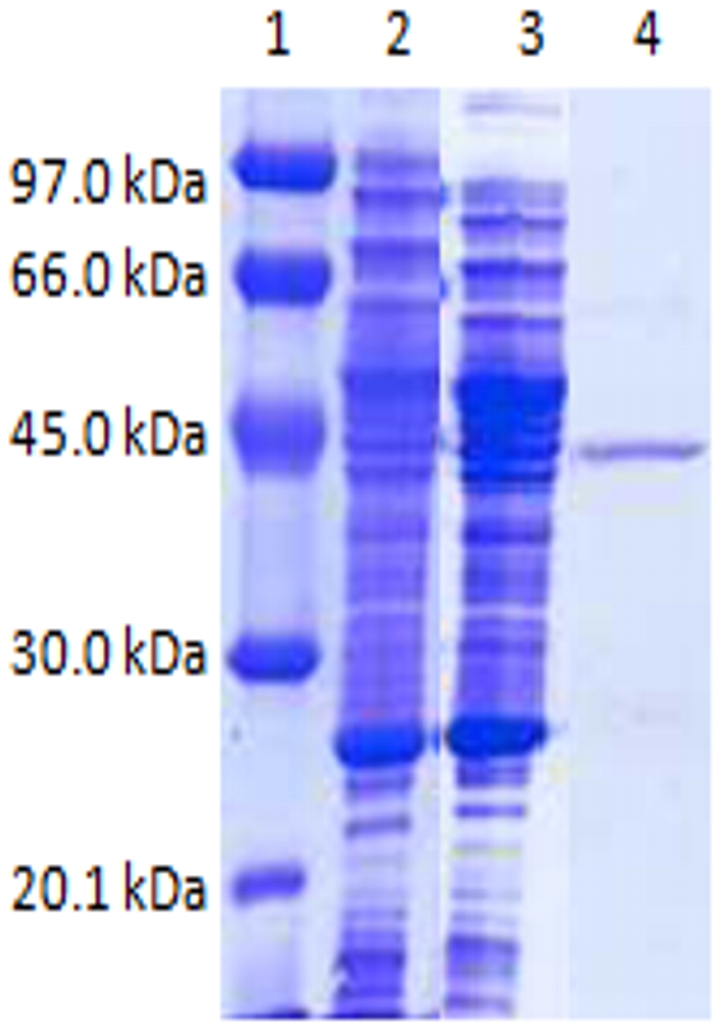
Characterization of LipBL by SDS-PAGE after purification from *E. coli* clone pFP. SDS-PAGE 12%. Lane 1, molecular standards; lane 2, *E. coli* crude extract expressing pBC KS vector; lane 3, *E. coli* crude extract expressing pFP plasmid; lane 4, LipBL after purification.

We used the clone *E. coli* pFP expressing LipBL to purify and characterize the soluble enzyme. The purification procedure that was carried out using different supports (ion exchange, chelates and hydrophobics) described in detail on Materials and Methods, led us to obtain an electrophoretically pure enzyme (Figure 3), with a purification yield of 60% and a purification factor higher than 100. The intrinsic activity of the pure enzyme was 50 U/mg when using p-nitrophenyl butyrate as substrate (Table 1). This enzyme showed a molecular mass of approximately 45 kDa (Figure 3).

**Table 1:** Purification of LipBL.

Protocol	Volume (ml)	Protein (mg/ml)	Total activity (U)	Specific activity (U mg^−1^)	Yield (%)	Purification factor
Crude extract	10	1.7	17.5	0.47	100	1
1	10	1.0	17.0	1.7	97.1	3.6
2	10	0.14	15.8	11.3	90.3	24.0
3	5	0.11	14.2	25.8	81.1	54.9
4	3	0.07	10.5	50.0	60.0	106.4

1: Incubation the *E. coli*/pFP crude extract in presence of sulfopropyl agarose.

2: Incubation the *E. coli*/pFP crude extract in presence of dextran-sulfate agarose and elution with 350 mM NaCl.

3: Incubation the *E. coli*/pFP crude extract in presence of IDA-Cu, elution with 100 mM imidazole and dialysis.

4: Incubation the *E. coli*/pFP crude extract in presence of octyl agarose, elution with 0.3% of Tritón X-100 and dialysis.

In order to confirm that the purified protein was LipBL we performed a peptide mass fingerprint (PMF) spectral analysis on the target protein on SDS-PAGE. The PMF spectrum of fragments of purified protein derived through trypsin digestion showed that the expressed protein presented an amino acid sequence identical to the sequence of LipBL, confirming that the protein was correctly expressed in *E. coli* (Figure S5).

### Biochemical characterization of LipBL

**Figure 4:**
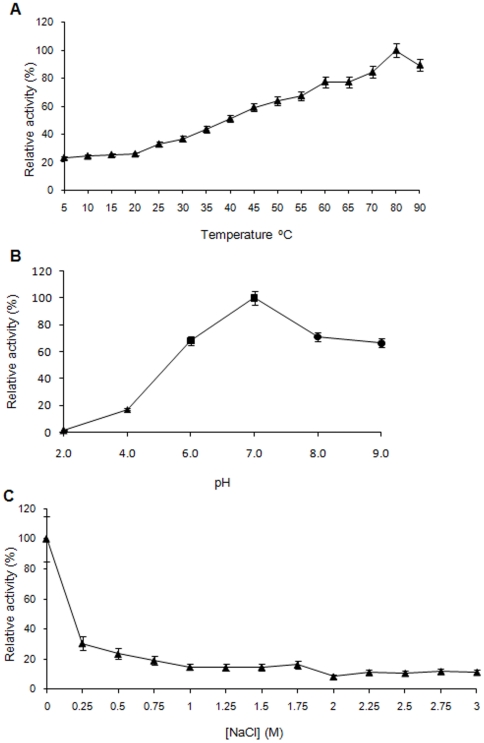
Effect of temperature, pH and NaCl concentration on the LipBL activity using p-NP butyrate as substrate. a) Effect of temperature on the activity of pure soluble LipBL at pH 7.0. b) Effect of pH at 25°C on the activity of the pure soluble LipBL. For the experiments, 50 mM of the following buffers were used: sodium acetate buffer for pH values of 2.0 and 4.0 (triangle), sodium phosphate buffer for pH values of 6.0 and 7.0 (square) and sodium bicarbonate buffer for pH values of 8.0 and 9.0 (circle). c) Effect of concentration of NaCl on the activity of LipBL at 25°C and pH 7.0. The lipolytic activity was determined following the method of Bastida et al. (1998) [12], using p-NP butyrate as substrate.

In order to determine the temperature, pH and NaCl ranges for optimum activity, the activity of the soluble LipBL was tested in different conditions. In the case of temperature, we studied a range of 5–90°C (Figure 4a). Results indicated that LipBL presents the optimal activity at 80°C, being active in a wide range of temperatures. On the other hand, the activity of LipBL was investigated over a pH range of 2.0–9.0 (Figure 4b). Results indicated that this enzyme presents optimal activity at pH 7.0. In the case of the NaCl studies, we investigated the activity over a NaCl range from 0 to 3 M (Figure 4c). The results obtained indicated that LipBL showed optimal activity in absence of salt, retaining 20% of activity in a wide range of NaCl concentrations (Figure 4c).

### Stability of LipBL to different temperatures and organic solvents

**Figure 5:**
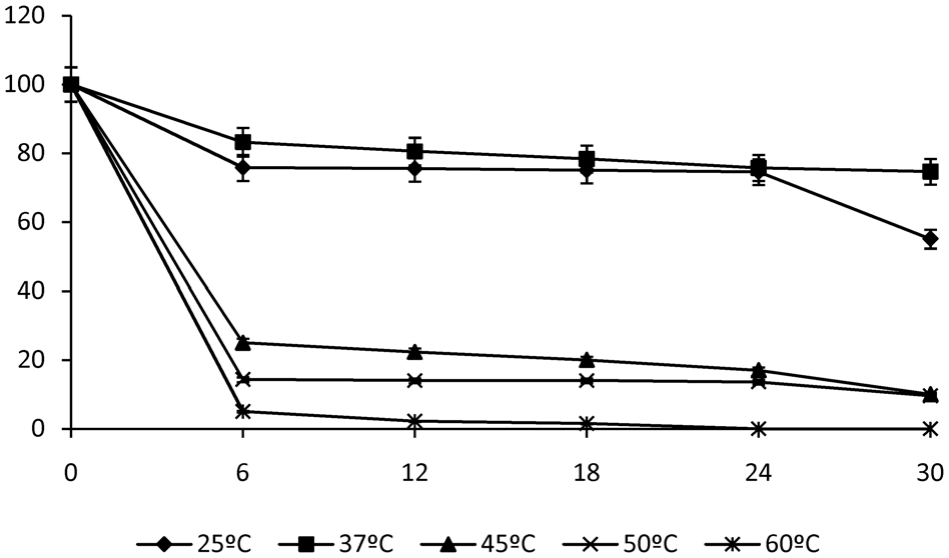
Thermostability of LipBL to different temperatures at pH 7. The lipolytic activity was determined following the method of Bastida et al. (1998) [12], using p-NP butyrate as substrate.

Thermostability data of pure soluble LipBL showed a 35% decrease in activity after one hour at 45°C, a 80% decrease after two hours at 50°C, and a 90% decrease after one hour at 60°C (Figure 5), however, this enzyme remains stable at 25°C and 37°C.

The stability of LipBL to different solvents was examined by standard assay techniques with the enzyme incubated in various buffer-solvent mixtures for 30 min, being quite stable in both high log P (hydrophobic) and low log P solvents (less hydrophobic and hydrophilic solvents) (Table 2). According to Laane et al. [30], solvents with high log P values cause less inactivation of biocatalysts than solvents with lower log P values. The results showed that methanol increased the stability of LipBL after 30 minutes, constituting the best solvent to be used in synthesis reactions. The other organic solvents at the concentrations tested maintained or slightly increased the activity of LipBL, except acetonitrile and 1-propanol (Table 2). The stability of LipBL in mixtures of aqueous buffer and organic solvents suggests that this enzyme resists denaturation by organic solvents and thus could be used in organic synthesis reactions.

**Table 2:** Effect of different organic solvents on the stability of the lipase LipBL.

Organic solvent	Log P^a^	Residual activity (%)^b^
Dimethylsulfoxide (DMSO) (30%)	−1.300	97.5±2.9
N,N-dimethylformamide (30%)	−1.000	98.3±2.9
Methanol (30%)	−0.760	120.5±3.6
Acetonitrile (30%)	−0.330	27.7±0.8
Ethanol (30%)	−0.240	107.8±3.2
Acetone (30%)	−0.230	91.3±2.7
2-Propanol (30%)	0.074	98.4±2.9
1-Propanol (30%)	0.280	35.1±1.0
Diethylether (30%)	0.850	115.1±3.4
Toluene (5%)	2.500	104.8±3.1
Hexane (5%)	3.500	94.1±2.8

a Log P values according to Laane et al. (1987) [30].

b Residual lipase activity toward p-nitrophenyl butyrate was measured after incubation of the protein with the solvent for 30 min. The activity in sodium phosphate buffer 25 mM after 30 min was considered 100%.

### Substrate selectivity and specificity of LipBL

**Figure 6:**
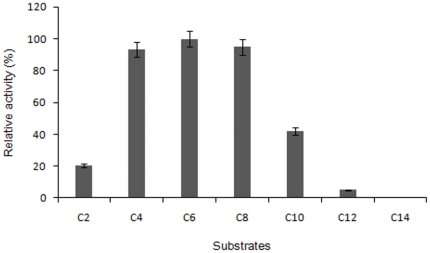
Activity of LipBL toward chromogenic substrates (*p*-nitrophenols) with different acyl chain lengths (acetate, C2; butyrate, C4; caprylate, C8; decanoate, C10; laurate, C12; myristate, C14).

The hydrolytic activity of LipBL against different fatty acid esters was investigated using a range of *p*-nitrophenyl (*p*-NP) esters at concentrations of 1 mM in dimethyl sulfoxide (DMSO) (acetate, C2; butyrate, C4; hexanoate, C6; caprylate, C8; decanoate, C10; laurate, C12 and myristate, C14). The LipBL hydrolytic pattern against *p*-NP esters (Figure 6) showed a strong preference towards short-medium length acyl chain, with *p*-NP hexanoate (C6) being the most easily hydrolyzed substrate. The hydrolytic activity of LipBL against long chain esters decreased drastically (Figure 6).

**Figure 7:**
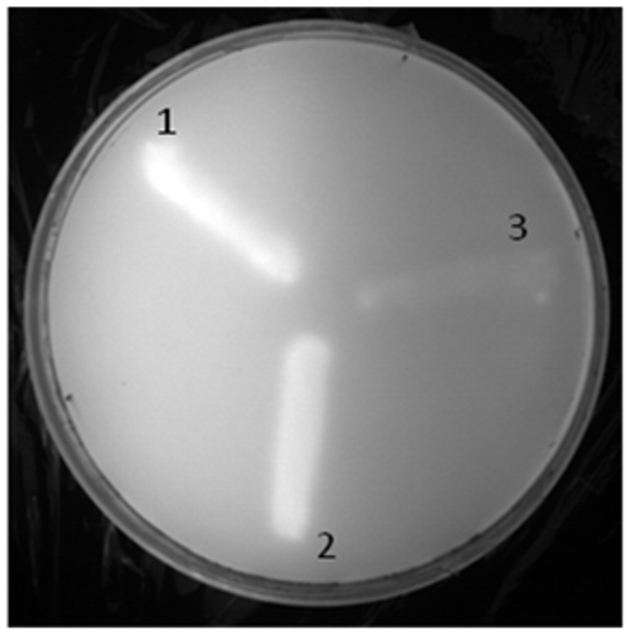
Plate assay for detection of hydrolysis of olive oil. 1. *E. coli* pFP clone (LipBL), 2. *Marinobacter lipolyticus* SM19 and 3. *E. coli* DH5α (negative control).

On the other hand, in order to study the hydrolysis of olive oil by LipBL, an in vitro plate assay was carried out, showing an orange fluorescence by exposure to UV light after 16 hours of incubation, indicating hydrolysis of olive oil [25] (Figure 7).

### Immobilization of LipBL in different supports

The immobilization of LipBL on different supports should improve the performance of this lipase in industrial reactors allowing us a continuous use and reuse of such interesting biocatalysts. Therefore, we construct LipBL-derivatives using supports with different properties such as octyl agarose (hydrophobic support), dextran-sulfate support (anionic support) and CNBr support, in which, the enzyme is covalently linked. The immobilization efficiency of LipBL for octyl agarose support after 30 minutes was 71.4±2.1%, obtaining a derivative showing a hydrolytic activity of 23.75±0.7 U g^−1^ using p-NPB as substrate. In the case of dextran sulfate support, the immobilization efficiency of LipBL after 1 hour was 36.44±1.1%, obtaining derivatives showing activity of 47±1.4 U g^−1^ using p-NP butyrate as substrate. The immobilization efficiency of LipBL for CNBr support was 92.3±2.7% from the total of offer protein obtaining a derivative showing an activity of 12.6±0.4 U g^−1^ for p-NP butyrate.

The LipBL-dextran sulfate, octyl agarose and CNBr derivatives were used to study the enantioselectivity of LipBL to different chiral substrates and CNBr and octyl agarose derivatives were used to determine the ability of LipBL to hydrolyze fish oil.

### Hydrolysis of fish oil using LipBL-derivatives

Due to the physiological importance of n-3 polyunsaturated fatty acids, we studied the hydrolysis of fish oil by LipBL-octyl and CNBr derivatives. The LipBL-CNBr derivative proved to be more effective in EPA enrichment than LipBL-octyl agarose (Table 3). The fish oil used in the experiment contained 18.6±0.5% of EPA, increasing to 27±0.8% in the released fatty acids by the activity of the LipBL-CNBr derivative, representing an enrichment of 45.2±1.3%. DHA, however, was only enriched 3.95±0.1%. These results indicated that LipBL shows more selectivity to EPA, being a good candidate to be used at industrial scale for the production of fish oils enriched in PUFAs.

**Table 3:** Hydrolysis of fish oil using different immobilized derivatives of LipBL.

Derivative	Total hydrolysis (%)	EPA (%)	DHA (%)	EPA^a^/DHA^b^^,^ c	% Hydrolysis/g catalyst	p-NP butyrate (U/ g catalyst)
LipBL-CNBr	4.6±0.1	8.5±0.2	0.5±0.01	16.8±0.5	9.3±0.3	12.6±0.4
LipBL-Octyl agarose	1.5±0.04	1.5±0.04	0.5±0.01	3.15±0.1	4.3±0.1	23.7±0.7

a EPA: eicosapentaenoic acid.

b DHA: docosahexaenoic acid.

c EPA/DHA: corresponds to free fatty acids released by hydrolysis of sardine oil.

### Enantioselectivity of LipBL-derivatives

Lipolytic enzymes often show high enantioselectivity to prochiral substrates, therefore we studied the enantioselectivity of LipBL-dextran sulfate derivative against butyroyl mandelic acid, methyl mandelate, dimethyl phenyl glutarate and 4-phenyl-2-hydroxy ethyl butyrate. The highest enantioselectivity (R-2.3) of the enzyme was observed using dimethyl phenyl glutarate as substrate (Table 4). In addition, studies on the enantioselectivity of various derivatives of LipBL (Octyl and CNBr) towards this substrate showed that the binding of LipBL to these supports did not affect the enantioselectivity of the enzyme.

**Table 4:** Hydrolysis of different substrates using LipBL-dextran-sulfate derivative.

Substrate	pH	Time (h)	Conversion (%)	Vh (UI/g x10^−2^)^c^	A specific^d^ (U/mg)	E^e^
Butyroyl mandelic acid^a^	7.0	0.7	100	40.0±1.2	0.85±0.02	S-1.20
Methyl mandelate^b^	7.0	24.0	17	1.2±0.03	0.02±0.0006	R-1.43
Dimethyl phenyl glutarate^a^	7.0	18.0	75	2.0±0.06	0.04±0.001	R-2.30
4-Phenyl-2-hydroxy ethyl butyrate (HPBT)^a^	7.0	0.5	100	32.0±0.9	0.68±0.02	1

a [substrate]: 1 mmol, biocatalyst 0.2 g, NaH_2_PO_4_ 2 ml, 25°C.

b [substrate]: 10 mmol, biocatalyst 0.2 g, NaH_2_PO_4_ 2 ml, 25°C.

c Calculated at 15–30% yield.

d Activity calculated for each p-NPB Unit/g derivative.

e Enantioselectivity value.

## Discussion

Extremozymes constitute a group of proteins of great biotechnological interest due to their stability in extreme conditions, opening new possibilities for different industrial processes that run in severe conditions of temperature, pH and pressure or in presence of organic solvents [31].

The presence of multiple lipolytic enzymes were detected in the intracellular fraction of the halophilic bacterium *Marinobacter lipolyticus* SM19. From this fraction, we have isolated and characterized a single lipolytic enzyme LipBL, showing high specific lipase activity (50 U/mg of protein by using p-nitrophenyl butyrate as substrate) and excellent response to different pH-values and temperatures. LipBL shows advantages over other non-halophilic and halophilic lipases, in particular the finding that the enzyme was active over a wide range of pH values (from 6.0 to 10.0) and temperatures (from 5 to 90°C) with maximum hydrolytic activity at pH 7.0 and 80°C. LipBL was quite stable for a large number of solvents for 30 minutes, including those with low log P that generally are considered to facilitate enzyme denaturation because they distort the water-biocatalyst interactions and strip the essential water from the enzyme (Table 2). Therefore, this enzyme could be used for a wide range of industrial processes. In spite of being an enzyme produced by a halophilic microorganism, this lipase is more active at low ionic strength but it preserves a relevant percentage of activity (around 20%) even in the presence of 3 M NaCl. It is important to highlight that halophilic enzymes are interesting for industrial applications due to their resistance to high salt concentrations, their extreme resistance to organic solvents and their thermostability [32].

Enzymes are usually costly and easy to inactivate in their soluble forms. Therefore, their immobilization is necessary in order to optimize their performance in industrial applications. Different supports have been used to obtain LipBL derivatives, which have been tested in regio- and enantioselective reactions. In the enantioselectivity studies, LipBL derivatives hydrolyze efficiently all prochiral (dimethyl phenyl glutarate) and chiral substrates tested (butyroyl mandelic acid, methyl mandelate, and 4-phenyl-2-hydroxy ethyl butyrate), although shows low enantioselectivity towards them (Table 4), in comparison with other lipases that hydrolyze only very specific substrates and show high enantioselectivity [33]. Moreover, the binding of LipBL to different supports did not affect the enantioselectivity of this enzyme, in contrast to other lipases previously studied [34]. On the other hand, we show the excellent behavior of LipBL derivatives in the production of free polyunsaturated fatty acids (PUFAs). The essential PUFAs comprise two main classes: n-6 and n-3 fatty acids and it has been proven that these fatty acids play important roles in pregnancy, lactation and childhood [35]. Furthermore, the anti-inflammatory properties of PUFAs could improve symptoms in diseases such as rheumatoid arthritis or asthma [36] and some studies suggest that PUFAs could prevent or reduce the progression of certain types of cancer [37], [38]. EPA (eicosapentaenoic acid) and DHA (docosahexaenoic acid) are normally obtained from fish oil as byproducts of processing it [39]. These oils contain about 30% of omega-3 PUFAs in combination with different fatty acids, mainly in the form of triglycerides. Several techniques have been developed for the purification of PUFAs from these oils using chemical or enzymatic methods [40]. The chemical methods present important disadvantages [40]. Therefore, a preferred method is the selective enzymatic hydrolysis of oils, leading to enrichment of PUFAs in the released free fatty acids by selective hydrolysis using a lipase [40], [41]. Unfortunately there are very few lipases that carry out this process efficiently. Our results indicate that the LipBL-CNBr derivative selectively releases EPA from fish oil, resulting in an enrichment of 45% in the released free fatty acids (Table 3). Tanaka *et al*. [42] described six lipases that hydrolyze tuna oil, finding that the lipase produced by *Candida cylindracea* presented low activity against esters of DHA, by increasing the concentration of this fatty acid in the form of glyceride and not producing increase of EPA. *Aspergillus niger* lipase [43] increased not only the DHA content but also EPA, although the absolute levels of this fatty acid released was quite low. The lipase from *Bacillus licheniformis* MTCC 6824 showed a high selectivity to increase EPA content in sardine oil (from 46.29 to 55.38%), although produces also an increase in DHA (from 4.02 to 5.08%). This enzyme is potentially useful to enrichment fish oil in omega-3 fatty acids [44].

## **Conclusions**

We have isolated and characterized a hydrolytic extremozyme, LipBL isolated from the halophilic bacterium *M. lipolyticus* SM19. This enzyme shows excellent biochemical properties (maximal activity at pH 7, 80°C, and in absence of sodium chloride), highlighting its great stability in presence of organic solvents.

LipBL was immobilized in different supports and tested the regio- and enantio-selective properties of the derivatives constructed. The LipBL-BrCN derivative showed a high efficiency in the enrichment of fish oil in free EPA. Compared to chemical methods, the use of lipases in the production of PUFAs presents enormous advantages, thus, LipBL is of great interest in the food industry.

## Supporting Information

Figure S1 **Asymmetric hydrolysis of dimethyl 3-phenylglutarate (1) by immovilized LipBL preparations.**
(TIFF)

Figure S2 **Enantioselective hydrolysis of methyl mandelate (4) by immovilized LipBL preparations.**
(TIFF)

Figure S3 **Enantioselective hydrolysis of 2-O-butyroil mandelic acid (5) by immovilized LipBL preparations.**
(TIFF)

Figure S4 **Enantioselective hydrolysis of 4-phenyl-2-hydroxyethylbutyrate (7) by immovilized LipBL preparations.**
(TIFF)

Figure S5 **MALDI-TOF Peptide Mass Fingerprinting (PMF) spectrum.** The PMF analysis was carried out on fragments of lipase LipBL obtained through trypsin digestion. The sequence coverage of these fragments is shown underlined.
(TIFF)

Table S1 **Similarity values of amino acid sequences for LipBL and homologue proteins**
(DOC)
