# Supplementary figures and images for: A Novel Halophilic Lipase, LipBL, Showing High Efficiency in the Production of Eicosapentaenoic Acid (EPA)
Source: PLoS One. 2011 Aug 10;6(8):e23325. doi: 10.1371/journal.pone.0023325 (PMC3154438; doi:10.1371/journal.pone.0023325)

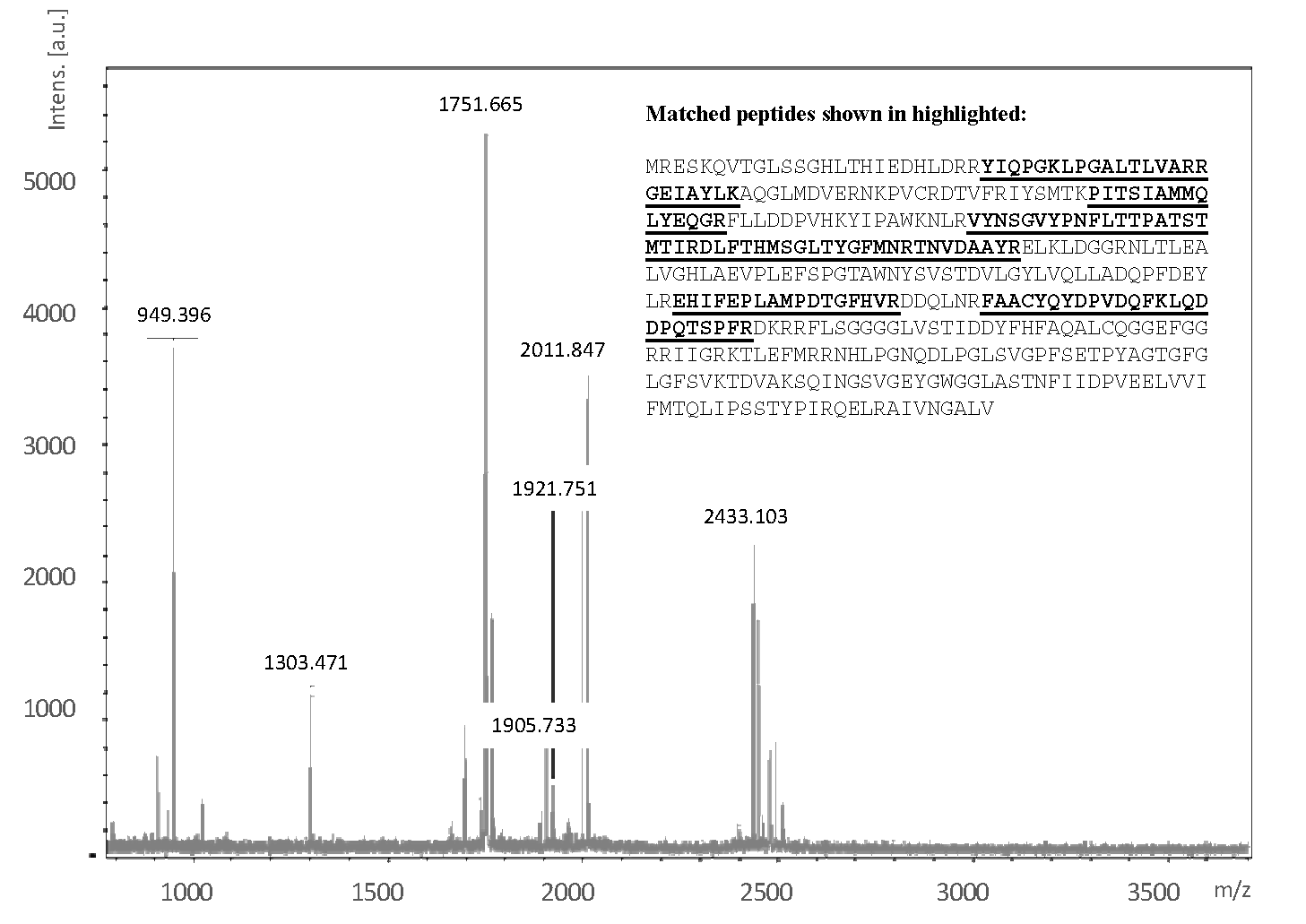

Supplement: Figure S5 — MALDI-TOF Peptide Mass Fingerprinting (PMF) spectrum. The PMF analysis was carried out on fragments of lipase LipBL obtained through trypsin digestion. The sequence coverage of these fragments is shown underlined. (TIFF) [file pone.0023325.s005.tiff]
