# Supplementary material for: A Novel Halophilic Lipase, LipBL, Showing High Efficiency in the Production of Eicosapentaenoic Acid (EPA)
Source: PLoS One. 2011 Aug 10;6(8):e23325. doi: 10.1371/journal.pone.0023325 (PMC3154438; doi:10.1371/journal.pone.0023325)
Supplement: Table S1 — Similarity values of amino acid sequences for LipBL and homologue proteins (DOC) [file pone.0023325.s006.doc]

Table S1 Similarity values of amino acid sequences for LipBL and homologue proteins

| Protein^a^ | Source^a^ | GenBank accession no. ^a^ | % Identity/similarity | E-value | Percent coverage (%) |
| --- | --- | --- | --- | --- | --- |
| LipBL | *Marinobacter lipolyticus* | CBX87546.1 |  |  |  |
| β-lactamase | *Marinobacter* sp. ELB17 | ZP_01736890.1 | 88/91 | 0.0 | 100 |
| β-lactamase | *Marinobacter aquaeolei* VT8 | YP_959634.1 | 74/83 | 6e-159 | 91 |
| β-lactamase | *Rhodopseudomonas palustris* BisB5 | YP_569345.1 | 55/68 | 5e-125 | 99 |
| Esterase | Uncultured bacterium | ACH88047.1 | 42/58 | 7e-73 | 94 |

^a^The corresponding protein, source, and database accession number are given for the some homologues to LipBL identified by a BLAST search at the NCBI. The protein and strain names of the microorganisms are given as originally designated by the authors.
